# Geospatial association of endemicity of ataxic polyneuropathy and highly cyanogenic cassava cultivars

**DOI:** 10.1186/1476-072X-12-41

**Published:** 2013-09-14

**Authors:** Olusegun Steven Ayodele Oluwole, Adeyinka Oludiran

**Affiliations:** 1 Neurology Unit, College of Medicine, University of Ibadan, Ibadan, Nigeria; 2 Family Health International, Abuja, Nigeria, Formerly, International Institute of Tropical Agriculture, Ibadan, Nigeria

**Keywords:** Geospatial, Cassava, Endemic, Cyanide, Ataxia, Polyneuropathy

## Abstract

**Background:**

Exposure to cyanide from cassava foods is present in communities where ataxic polyneuropathy is endemic. Ataxic polyneuropathy is endemic in coastal parts of southwest and southeast Nigeria, and coastal Newala, south India, but it has been reported in epidemic or endemic forms from Africa, Asia, or Caribbean. This study was done to determine if cyanogenicity of cassava cultivars is higher in lowland than highland areas, and if areas of endemicity of ataxic polyneuropathy colocalize with areas of highest cyanogenicity of cassava.

**Methods:**

Roots of cassava cultivars were collected from 150 farmers in 32 of 37 administrative areas in Nigeria. Global positioning system was used to determine the location of the roots. Roots were assayed for concentrations of cyanogens. Thin Plate Spline regression was used to produce the contour map of cyanogenicity of the study area. Contour maps of altitude of the endemic areas were produced. Relationship of cyanogenicity of cassava cultivars and altitude, and of locations of areas of high cyanogenicity and areas of endemicity were determined.

**Results:**

Geometrical mean (95% CI) cyanogen concentration was 182 (142–233) mg HCN eq/kg dry wt for cassava cultivars in areas ≤ 25 m above sea level, but 54 (43–66) mg HCN eq/kg dry wt for areas > 375 m. Non-spatial linear regression of altitude on logarithm transformed concentrations of cyanogens showed highly significant association, (p < 0.0001). Contour map of concentrations of cyanogens in cassava cultivars in Nigeria showed four areas with average concentrations of cassava cyanogens > 250 mg HCN eq/kg dry wt, and one area of moderately high cyanogen concentration > 150 mg HCN eq/kg dry wt. The endemic areas colocalized with areas of highest cassava cyanogenicity in lowland areas close to the Atlantic Ocean.

**Conclusion:**

This study shows strong geospatial association of areas of endemicity of ataxic polyneuropathy and areas of highest cyanogenicity of cassava cultivars. Finding of higher cyanogenicity of cassava in lowland than highland areas indicate strong influence of altitude on expression of cyanogens in cassava cultivars.

## Background

Occurrence of ataxic polyneuropathy has been attributed to exposure to cyanide from cassava foods [1-4]. Ataxic polyneuropathy is a neurological syndrome of distal symmetrical sensory polyneuropathy, sensory gait ataxia, optic atrophy, and neurosensory deafness [4,5]. Epidemic and endemic forms have been described from the Caribbean [6-8], Africa [4,5,9], and Asia [10] for more than 100 years. In Nigeria, endemic ataxic polyneuropathy was mapped to two geographical areas, in the southwest and southeast in the 1950s [11] and 1960s [4,12], but occurrence was low outside of these regions [13]. It was shown in the early 2000s [5] that occurrence of ataxic polyneuropathy persists in parts of the endemic area, although all communities in the previously defined endemic areas have not been re-surveyed. Cassava supplied more than two thirds of dietary calories in the southwest endemic area in surveys of the 1960s [12,14] and of 2000s [15,16]. It has also been shown that consumption of cassava foods in the endemic communities was more than twice that of non-endemic communities [17]. Although it has been postulated that cyanogenicity of cassava cultivars is high in the endemic areas, it has not been shown that cassava cultivars of highest cyanogenicity are present only in the endemic areas.

Cassava, *Manihot esculenta* Crantz, is a short-lived perennial shrub [18] which grows between latitude 30°N and 30°S of the equator where mean temperature is greater than 18°C [19]. It is described as a food security crop because it is well adapted to grow in drought prone environment or in poor soil [20]. Unlike major food crops like rice, *Oryza spp*., maize, *Zea spp*., and wheat, *Triticum spp*., which are cyanogenic only during development [21,22], cassava stores cyanogenic glycosides in its edible roots. All cassava cultivars contain two cyanogenic glycosides, linamarin [23,24] and lotaustralin [23,25], which are present in a ratio of 97:3 [23]. Field [3,15] and experimental studies [26,27] have shown that meals from cassava products cause exposure to cyanide. Snacks of cassava have also been shown in Australia to contain very high concentrations of cyanogens [28]. Although studies of the 1950s and 1960s attributed occurrence of endemic ataxic polyneuropathy to exposure to cyanide from cassava foods, studies of the early 2000s [16,29] did not find evidence of association. Ataxic polyneuropathy, however, remains endemic in localized areas of coastal Nigeria, and coastal Newala, south India where cassava is staple. This study was done to determine if cyanogenicity of cassava cultivars is higher in lowland than highland areas, and if there is geospatial colocalization of areas of highest cyanogenicity of cassava cultivars and areas of endemicity of ataxic polyneuropathy.

## Methods

### Sampling of cassava cultivars

Nigeria, the most populous African country, is located between longitudes 3.0–14°E, and latitudes 3.0–14°N. Its major administrative areas are the 36 states and the Federal Capital Territory. It was not feasible to divide the country into a regular grid and sample cassava cultivars from each grid, because cassava farms were not randomly sited. The administrative areas were, therefore, used as polygons within which cassava cultivars were sampled. The Ministry of Agriculture of administrative areas, farmers associations, and cassava processors were contacted to know where cassava farms were in each state, and all the locally named cultivars. Cassava roots were sampled from all named cultivars. The spatial location of all cassava cultivars, longitude, latitude, and altitude, were determined using the Magellan global positioning system.

### Processing and determination of cyanogens in cassava roots

Cassava roots, which were harvested by the farmers, were processed for storage within 12 hours of harvesting. The roots were peeled and cut into longitudinal slices. Cubes of 1 cm sides were diced from the longitudinal slices. 15 g of diced roots, which were weighed into 250 ml cold 0.1 M orthophosphoric acid, were homogenized using a Waring blender. Homogenates were centrifuged, and triplicates of supernatants were stored on ice on the fields, and transported to the laboratory for storage at −80°C until assayed. Determination of cyanogenic compounds in the stored supernatants were done in triplicates using an improved enzymatic method [30].

### The endemic areas

The endemic communities of Ososa, Ibefun, Igbile, Idowa, Omu, Ejinrin, Epe, and Iraye [12], which lie at geographical boundaries of 3.4°–4.0°E and 6.6°–6.7°N in southwest Nigeria, were defined in 1964. The area of endemicity in southeastern Nigeria was described as Sapele area [11] in 1955. The city of Sapele is located at 5.7°E, 5.9°N. The city of Udo, another community where ataxic polyneuropathy has been described [31], is located at 5.4°E, 6.5°N.

### Maps

Concentrations of cyanogens in cassava roots were grouped into quartiles, and plotted on a topographical map of Nigeria using the Generic Mapping Tools GMT version 4.5.9, 2013 [32]. Cassava cyanogens of the first quartile were coloured blue, the second quartile coloured green, the third quartile coloured yellow, and the fourth quartile coloured red. Contour map of cyanogenicity was produced using the field package of R Statistical Programming and Environment, Vienna, Austria, version 3.0.1, 2013 [33]. Contour maps of altitude above the sea level were produced, using the GMT version 4.5.9, 2013 [32], to determine the elevation of southwestern and southeastern endemic areas relative to that of non-endemic areas.

### Statistics

Non-spatial linear regression analysis of quartiles of altitude on log(cyanogen) was performed. Thin plate spline regression, one of several methods of spatial interpolation, for he used to produce the contour map of cyanogens, because it is based on deterministic theory. Concentration of cyanogens in cassava roots, in mg HCN eq per kg dry wt, was the dependent variable while the spatial location was the independent variable. Altitude was included as covariate. All statistical analyses were done, using the R Statistical Programming and Environment, Vienna, Austria, version 3.0.1, 2013 [33].

## Results

### Cassava cultivars and cyanogenicity

**Figure 1:**
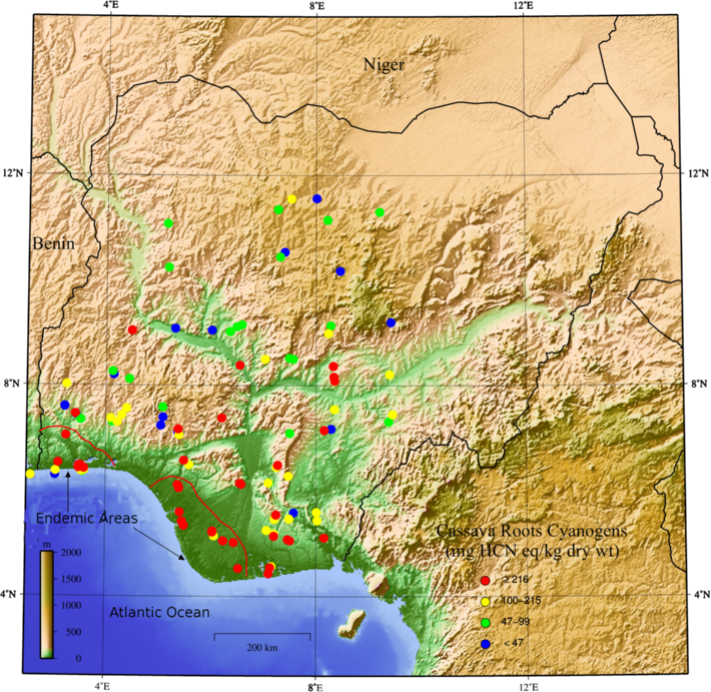
Topographical map of nigeria showing distribution of concentrations of cassava cyanogens.

Cassava cultivars were collected from 150 farmers in 32 of 37 major administrative areas of the Federal Capital Territory in Nigeria; 47 farmers in the southwest Nigeria, 48 farmers in the southeast Nigeria, and 55 farmers in northern Nigeria. Total 421 cassava roots were collected, of which only 20 (5%) had names linked to Agricultural Institutes. There were 251 unique names in the three geographical areas; 60 in the southwest, 115 in the southeast, and 126 in the north. Names of cassava cultivars were similar within language areas of northern, southwestern and eastern Nigeria, with few overlaps between language areas. The concentrations of cyanogens in cassava cultivars are shown in the topographical map of Nigeria, (Figure 1).

**Figure 2:**
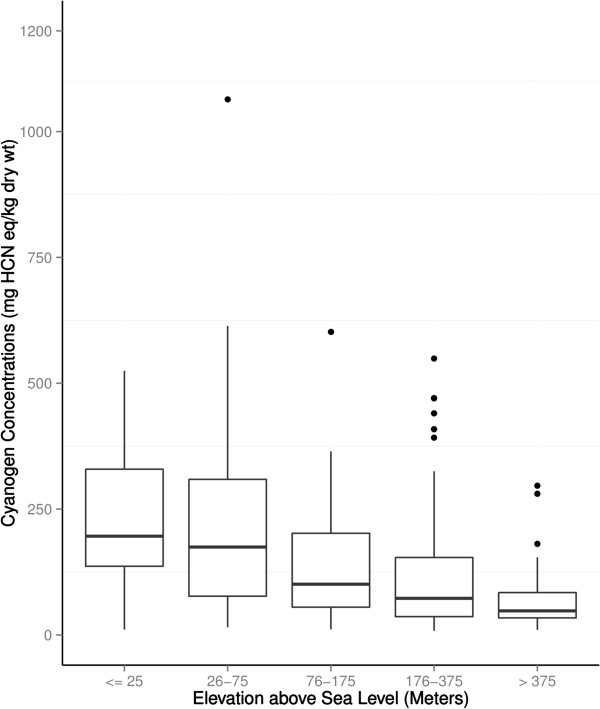
Boxplot of altitude and cassava cyanogenicity.

Geometrical mean (95% CI) cyanogen was 182 (142–233) mg HCN eq/kg dry wt for cassava cultivars in areas ≤ 25 m above sea level, but 54 (43–66) mg HCN eq/kg dry wt for areas > 375 m. Boxplots of concentrations of cyanogens in cassava cultivars, grouped by altitude above the sea level, is shown in Figure 2. Geometrical mean cyanogen was 101 mg HCN eq/kg dry wt (range 8–1064) for all cassava cultivars, 182 mg HCN eq/kg dry wt (range 15–1064) for cassava cultivars in the endemic area of southwest, but 60 mg HCN eq/kg dry wt (15–281) for those from non-endemic southwestern Nigeria, 127 mg HCN eq/kg dry wt (8–614) for those from the southeastern Nigeria, and 80 mg HCN eq/kg dry wt (8–614) for those from northern Nigeria. Non-spatial linear regression of altitude on logarithm transformed concentrations of cyanogens showed highly significant association, (p < 0.0001).

### Areas of high cyanogenicity

**Figure 3:**
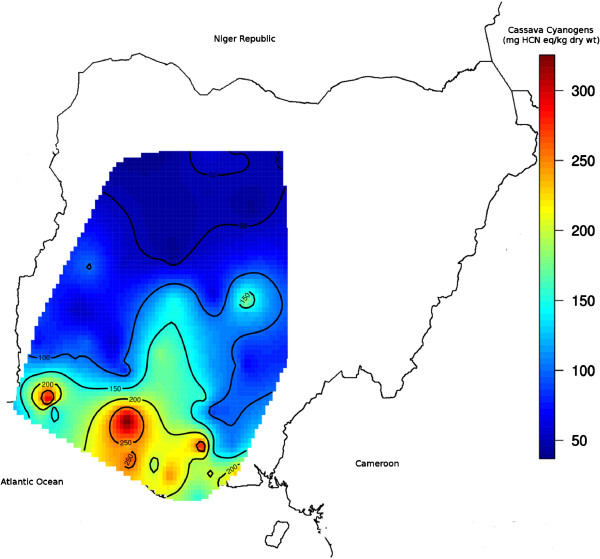
Contour map of cassava cyanogenicity.

The contour map of concentrations of cyanogens in cassava cultivars in Nigeria showed four areas with average concentrations of cassava cyanogens exceeding 250 mg HCN eq/kg dry wt, and one area of moderately high cyanogen concentration exceeding 150 mg HCN eq/kg dry wt, (Figure 3). The area of high cyanogenicity in the southwest centred on 3.5°E, 6.5°N; while the areas in the southeast centred on 5.5°E, 6.0°N; 5.5°E, 5.2°N; and 7.2°E, 5.5°N. The area of moderately high cyanogenicity centred on 8.2°E, 8.3°N in the river Benue basin.

### High cyanogenicity and endemic areas

**Figure 4:**
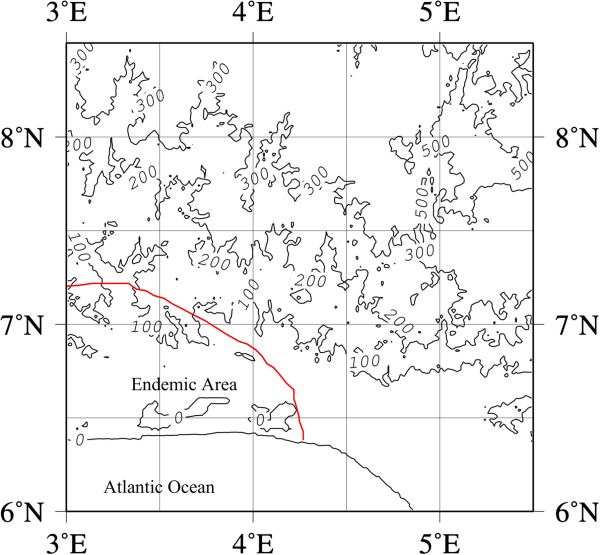
Contour map of altitude of southwest endemic area.

**Figure 5:**
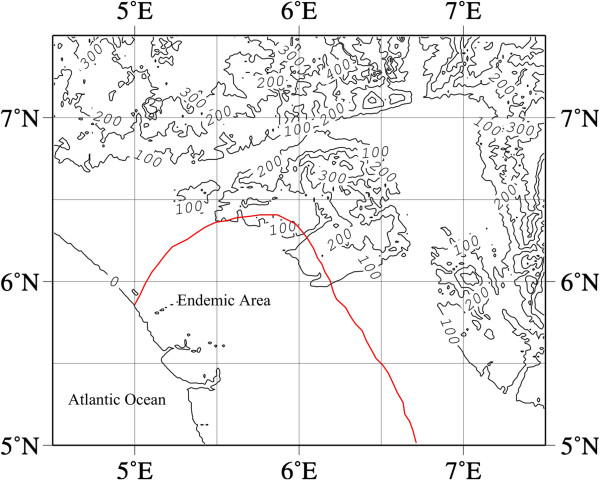
Contour map of altitude of southeast endemic area.

The endemic area of southwest Nigeria, fell entirely within the area of high cyanogenicity in southwestern Nigeria, while the endemic area of southeastern Nigeria fell entirely in the southeastern area of high cyanogenicity. The altitudes of the endemic areas of southwestern (Figure 4) and southeastern (Figure 5) Nigeria were about sea level.

## Discussion

This study shows that both endemic areas of southwestern and southeastern Nigeria colocalized with areas of highest cassava cyanogenicity, which are in lowland areas close to the Atlantic Ocean. Endemic ataxic polyneuropathy was reported from coastal and lowland Jamaica in 1897 [6], but another report of endemic ataxic polyneuropathy from Jamaica, Trinidad, Barbados, Montserrat, Antigua, and El Salvador was published in 1964 [7]. Epidemic ataxic polyneuropathy, which occurred in a sugar cane plantation, was reported from Jamaica in 1918 [34]. In Cuba, an epidemic of optic neuropathy and polyneuropathy occurred between 1991 and 1994 [8]. Occurrence was highest in the western lowland area of Pinar del Rio Province. Cases have also been reported from Dar es Salaam, a coastal city in Tanzania [9,35], and from Kerala region, a coastal region in southwest India [10]. The endemic areas in Nigeria are also lowland areas close to the Atlantic Ocean. Thus, there is strong association of altitude and occurrence of epidemic and endemic ataxic polyneuropathy.

Occurrence of epidemic ataxic polyneuropathy of Jamaica has been linked to exposure to cyanide from sugar cane [1], while the epidemic ataxic polyneuropathy of Cuba has been linked to exposure to cyanide from cigars and cassava foods [2]. In Tanzania exposure to cyanide from cassava was found in cases of ataxic polyneuropathy in 1972 [35]. Occurrence of ataxic polyneuropathy in Kerala region of India [10] was also linked to exposure to cyanide from cassava foods. Studies of the southwestern Nigerian endemic area in the early 2000s [15,16] showed that cassava supplied more than two-thirds of dietary calories of the communities. Although, this study shows high cyanogenicity of cassava in the endemic area, the relationship of highly cyanogenic cassava cultivars and of exposure to cyanide is more complex since raw cassava is not commonly consumed. Thus, high exposure of endemic area population is a combination of high cyanogenicity of cultivars and inefficient methods of processing cassava roots.

Although cassava roots are eaten raw in farming communities, cassava is usually processed to products like flour, pastes, and granules, which have much longer shelf-life than raw cassava roots [15]. It has been shown that cassava roots with 2000 mg HCN eq/kg dry wt can be processed to products with less than 20 mg HCN eq/kg dry wt [36]. Although further reductions occur during preparation of products like cassava flours, pastes, and granules to meals, sufficient cyanogens that can cause exposure to cyanide often remain [29]. An experimental study in Cameroon showed that concentrations of cyanogens in cassava roots were reduced from 197–511 mg HCN eq/kg dry wt to 5.6–14 mg HCN eq/kg dry wt in bâton de manioc, from 449–951 mg HCN eq/kg dry wt to 19–41 mg HCN eq/kg dry wt in *fufu*, and from 427–740 mg HCN eq/kg dry wt to 15–27 mg HCN eq/kg dry wt in *gari*[37]. Foods like bâton de manioc, which are prepared from paste like products, usually have lower concentrations of cyanogens than foods from cassava flours and granules [15]. Foods from cassava granules, locally called *gari*, are the most common cassava foods in the endemic areas of southwest and southeast Nigeria. Market surveys of *gari* showed that *gari* in the southwestern endemic area was more cyanogenic than those of other areas [15]. Experimental study showed that the method of processing cassava roots to gari in the endemic area of southwestern Nigeria was not efficient [29]. Thus, efficient cassava processing can produce relatively safe cassava foods, which have low concentrations of cyanogens.

Highly cyanogenic cassava cultivars have been found in the Tukanoans and other indigenous groups in lowland Amazonia [38]. Although selection of highly cyanogenic cultivars by farmers may explain this observation, this study shows that environmental factors are the most probable reason for the localized highly cyanogenic cultivars of the endemic areas, since similar cassava cultivars are planted in the endemic and non-endemic southwestern area. Environmental factors like rainfall, drought, and nitrogen content of the soil have been shown to increase or decrease the concentrations of cyanogens in cassava [39,40]. Thus, high cyanogenicity of cassava cultivars in the endemic areas is attributable to environmental rather than genotype factors.

Exposure to cyanide from cassava foods is linked to occurrence of konzo, a neurological syndrome of acute or subacute onset paraparesis or quadriparesis, spastic dysarthria, and nystagmus, which reach maximum disability in hours or a few days [41]. Resolution of speech and ocular deficits usually occur, but skeletomotor deficits often persist without appreciable improvement [41]. This neurological syndrome has been described in epidemic and endemic forms from Mozambique, Congo DR, Central African Republic, Tanzania, and Cameroon. Seasonal epidemics, which is intervened by yearly low occurrence, dominate the epidemiology of konzo [42]. The average altitude of Nampula district, Mozambique where konzo epidemic occurred in 1981 is 431 m [41]. In Congo DR, altitudes of Kikwit, Popokabaka, Kahemba, and Masi-Manimba, where konzo has been described are above 400 m. In Tanzania, altitudes of Mtanda, Kigoma, and Mwanza, where konzo has been described [43] are above 700 m. Thus, occurrence of konzo is not confined to lowland areas.

Alternative risk factors, other than exposure to cyanide, have been considered for both ataxic polyneuropathy and konzo. Deficiency of thiamine [44], vitamin B [45], and riboflavin [46] were also considered possible causal factors for ataxic polyneuropathy. Subjects with ataxic polyneuropathy and control subjects in Nigeria [47] and Cuba [48], however, had similar concentrations of thiamine. Clinical trials of vitamin B [45], riboflavine [46], and riboflavine and cystine [49] were unsuccessful in Nigerian subjects. No evidence of viral infections were found for ataxic polyneuropathy [15] and konzo [50].

## Conclusions

Endemic ataxic polyneuropathy was one of neurological syndromes that were described as hidden endemias in 1985 [1]. It remains endemic in southwestern Nigeria, more than 50 years after it was first described. This study, which to date sampled the widest geographical area in Nigeria, shows strong geospatial association of highly cyanogenic cassava cultivars and endemicity of ataxic polyneuropathy. It also shows, for the first time, that cyanogenicity of cassava is higher in lowland than highland areas. Since ataxic polyneuropathy is endemic in lowland areas, altitude may be an important environmental variable in the causation of this syndrome. Although breeding of low cyanogenic cultivars has been suggested as a public health strategy to reduce exposure to cyanide from cassava foods, the findings of this study indicate that breeding low cyanogenic cultivars is unlikely to achieve the stated objectives, since expression of cyanogenicity may increase in lowland areas.

## Competing interests

The authors declare that they have no competing interests.

## Authors’ contributions

The concept and design of the study, statistical analysis, and draft manuscript were prepared by OSAO; collection of cassava cultivars, assays for cyanogens, and revisions of manuscript were done by OSAO and AO IPICS played no role in study design, data collection and analysis, interpretation of data, writing of the manuscript, and the decision to submit the article for publication. Both authors read and approved the final manuscript.
